# Prevalence and Prognostic Significance of Malnutrition in Hypertensive Patients in a Community Setting

**DOI:** 10.3389/fnut.2022.822376

**Published:** 2022-02-23

**Authors:** Zhi-wen Yang, Xue-biao Wei, Bing-qi Fu, Ji-yan Chen, Dan-qing Yu

**Affiliations:** ^1^Shantou University Medical College, Shantou, China; ^2^Division of Cardiology, Guangdong Cardiovascular Institute, Guangdong Provincial Key Laboratory of Coronary Heart Disease Prevention, Guangdong Provincial People's Hospital, Guangdong Academy of Medical Sciences, Guangzhou, China; ^3^Division of Geriatrics Intensive Medicine, Guangdong Provincial Geriatrics Institute, Guangdong Provincial People's Hospital, Guangdong Academy of Medical Sciences, Guangzhou, China

**Keywords:** malnutrition, hypertension, Controlling Nutritional Status (CONUT) score, Nutritional Risk Index (NRI), Naples Prognostic Score, cardiovascular mortality, all-cause mortality

## Abstract

**Background:**

Malnutrition is a significantly poor prognostic factor for a variety of cardiovascular diseases. However, its prevalence and prognostic value in hypertensive patients is still unclear. The present study sought to determine the prevalence and prognostic value of malnutrition in hypertensive patients in a community setting.

**Methods:**

We included 9,949 hypertensive patients from the National Health and Nutrition Examination Survey (NHANES) (2005–2014). The Controlling Nutritional Status (CONUT) score, the Nutritional Risk Index (NRI), and the Naples Prognostic Score (NPS) were applied to assess the nutritional status of participants. A Cox regression model was established to examine the association between malnutrition and cardiovascular and all-cause mortality.

**Results:**

In all, 19.9, 3.9, and 82.9% hypertensive patients were considered to have malnutrition as evaluated by the CONUT, NRI, and NPS, respectively. Malnutrition assessed by CONUT and NRI was independently associated with cardiovascular mortality (HR [95% CI]) for mild and moderate-to-severe degree of malnutrition, respectively: 1.41 (1.04–1.91) and 5.79 (2.34–14.29) for CONUT; 2.60 (1.34–5.07) and 3.30 (1.66–6.56) for NRI (all *P* < 0.05), and for all-cause mortality (HR [95% CI]) for mild and moderate-to-severe degree of malnutrition, respectively: 1.48 (1.30–1.70) and 4.87 (3.40–6.98) for CONUT; 1.72 (1.24–2.39) and 2.60 (1.96–3.44) for NRI (all *P* < 0.01). Naples Prognostic Score could only independently predict all-cause mortality.

**Conclusions:**

Malnutrition was common among hypertensive patients and was closely associated with both long-term cardiovascular and all-cause mortality.

## Introduction

The effect of nutritional state on a variety of cardiovascular diseases is now the subject of increasing concern, as it is modifiable compared to other clinical variables (1). Most previous studies were focused on overnutrition and the results suggested that it was a significant risk factor for cardiovascular disease (2). However, recent studies have reported that malnutrition is a significantly poor prognostic factor of acute coronary artery disease, heart failure, atrial fibrillation, and valvular heart disease (3–5).

Hypertension, one of the most commonly occurring diseases worldwide (6), contributes to the risk of developing coronary heart disease, stroke, and other cardiovascular disease (7). The aged-standardized prevalence of hypertension reported on 2015 was 24.1% for men and 20.1% for women globally (8). Moreover, nutritional factors such as nutrient intake, blood lipids, and high Body Mass Index (BMI) have been shown to be associated with blood pressure control and mortality (9, 10). However, less attention has been paid to the prevalence and prognostic value of malnutrition among hypertensive patients.

Thus, we aimed to determine the prevalence and prognostic value of malnutrition among hypertensive patients in a community setting by using three nutritional screening tools (NSTs), namely Controlling Nutritional Status (CONUT), Nutritional Risk Index (NRI), and Naples Prognostic Score (NPS).

## Methods

### Study Population

**Figure 1:**
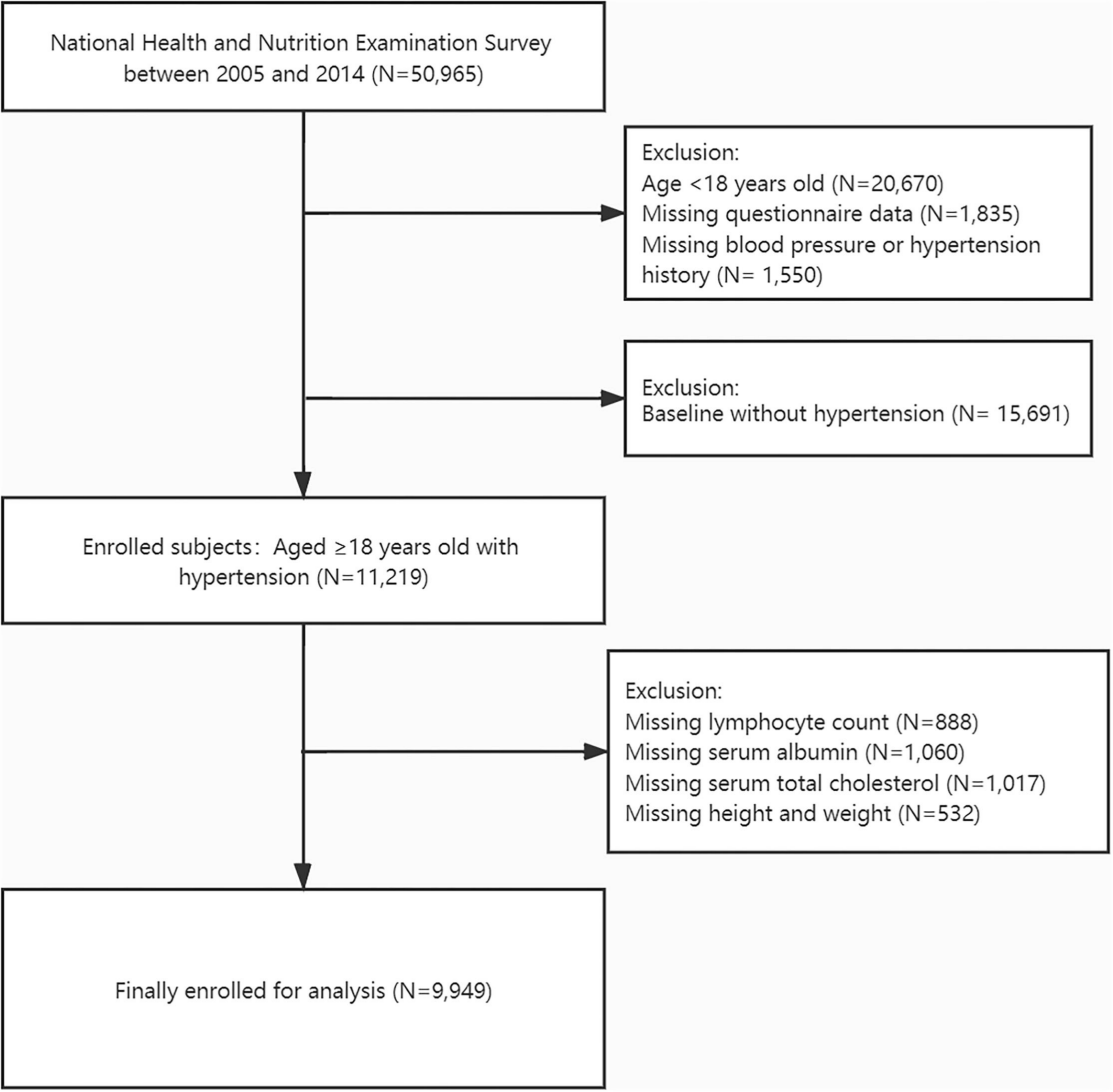
Flow diagram for the selection of the study population.

This retrospective observational study was based on National Health and Nutrition Examination Survey (NHANES) (2005–2014) (11)—a large nationwide survey on the civilian US population conducted by the National Center for Health Statistics of the Centers for Disease Control and Prevention. All NHANES study protocols survey protocol was approved by the Ethics Review Committee of NCHS of the Centers for Disease Control and Prevention. All participants had provided written, informed consent for the use of their data. All procedures in this study were conducted in accordance with all the relevant guidelines. We included participants aged ≥18 years with hypertension. However, individuals with missing data on lymphocyte count (*n* = 888), serum albumin (*n* = 1,060), serum total cholesterol (*n* = 1,017), and height and weight (*n* = 532) was excluded, leaving 9,949 participants for the final analysis (Figure 1).

### Baseline Assessment

The data on physical examination, questionnaires, and laboratory examination was obtained from NHANES, which were performed in a standardized manner. Covariates including sociodemographic information; current smoking status; current alcohol drinking, medical history (congestive heart disease, coronary heart disease, diabetes, stroke, emphysema, liver disease, and malignant tumor); BMI; hemoglobin; albuminuria; and estimated glomerular filtration rate (eGFR) were assessed. Body Mass Index was defined as the weight in kilograms divided by the square of height in meters. Albuminuria was defined by urinary albumin creatine ratio ≥30 mg/g and eGFR was calculated using the Chronic Kidney Disease Epidemiology Collaboration equation.

### Definition of Hypertension

Hypertension and antihypertensive medication history were collected by questionnaires. Blood pressure was obtained with a mercury sphygmomanometer with an appropriately sized cuff by a trained physician. Blood pressure measurement was performed three times and the average value of the three measurements was defined as the systolic blood pressure (SBP) and diastolic blood pressure (DBP). Hypertension was defined as having a self-reported hypertension history or using antihypertensive medications or SBP ≥140 mmHg or DPB ≥90 mmHg.

### Nutrition Status Assessment

The CONUT score (12), calculated based on the levels of serum albumin, total cholesterol, and lymphocytes, was developed as a screening tool for early detection of malnutrition. We categorized the scores into three groups: normal, 0–1; mild, 2–4; and moderate-to-severe, 5–12.

The NRI (13), a popular nutrition screening tool in recent years, was originally defined as 1.519 × serum albumin (g/l)+41.7 × (current body weight[kg]/usual body weight [kg]).The actual body weight was usually replaced by ideal body weight which is defined as height (cm) – 100 – ([height (cm) – 150]/2.5) for women and height (cm) – 100 – ([height (cm) – 150]/4) for men. Patients were categorized into three groups according to their NRI: no nutritional risk (NRI ≥ 100), mild nutritional risk (97.5 ≤ NRI <100), and moderate-to-severe nutritional risk (NRI <97.5).

The NPS (14), a tool to access the nutritional and inflammatory status of patients, is often used among patients with malignancies. The NPS takes into account serum albumin (mg/dl), total cholesterol (mg/dl), the neutrophil:lymphocyte ratio, and lymphocyte:monocyte ratio. A score of 0 is considered normal; scores of 1–2 and 3–4 reflect mild and moderate-to-severe malnutrition, respectively.

### Outcomes

The endpoints were long-term cardiovascular or all-cause mortality. The mortality status of participants was obtained by data matching with death certificates in the National Death Index until December 31, 2015. Cardiovascular death was determined based on the International Classification of Diseases, 10th Edition, Clinical Modification System codes (I00–I09, I11, I13, I20–I51).

### Statistical Analysis

Baseline characteristics were expressed as a median with interquartile range (25^th^-75^th^ percentiles) for continuous variables and with categorical data expressed as *n* (%). Venn diagrams were used to illustrate the relationship between the three malnutritional indices. Survival analysis was performed with standardized Kaplan–Meier curves and the log-rank test. Cox proportional hazards regression models were used to estimate hazard ratios (HRs) and 95% confidence intervals (CIs) for cardiovascular and all-cause mortality.

All statistical analyses were performed using SPSS v25.0 (IBM Corporation, Armonk, NY, USA) and eulerAPE v3 (15). A two-sided *P* < 0.05 was considered to indicate statistical significance.

## Results

### Baseline Characteristics of the Study Population

The baseline characteristics of participants are summarized in Table 1. The analysis included 9,949 hypertensive patients with a mean age of 49.1 ± 17.8 years; 50.1% subjects were female. Overall, 12.8% participants died during the survey with a mean follow-up time of 5.48 years; of these, 244 (2.5%) participants died from cardiovascular causes.

**Table 1:** Baseline clinical characteristics of included patients.

**Variables**	**Total (*n* = 9,949)**
Age, years	59.64 ± 15.20
Female gender, *n* (%)	4,992 (50.2)
Ethnicity, *n* (%)	
Non-white	4,750 (47.7)
White	5,199 (52.3)
BMI	30.6 ± 7.2
BMI classification	
Underweight	97 (1.0)
Normal	1,908 (19.2)
Overweight	3,251 (32.7)
Obesity	4,693 (47.2)
Current smoke, *n* (%)	1,896 (19.1)
Diabetes mellitus, *n* (%)	2,136 (22.1)
Stroke, *n* (%)	710 (7.2)
Emphysema, *n* (%)	321 (3.2)
Liver disease, *n* (%)	486 (4.9)
Malignant tumor, *n* (%)	1,367 (13.8)
Congestive heart failure, *n* (%)	608 (6.1)
Coronary heart disease, *n* (%)	763 (7.7)
Albuminuria, *n* (%)	1,949 (19.9)
Hemoglobin (g/L)	13.98 ± 1.58
Serum albumin (g/L)	41.84 ± 3.32
Lymphocyte count (10^9^/L)	2.12 ± 1.14
Total cholesterol (mmol/L)	5.06 ± 1.12
eGFR(ml/min/1.73 m^2^)	79.11 ± 23.00
Follow-up time, years	5.47 ± 2.82
Long-term mortality*, *n* (%)	
All-cause	1,254 (12.6)
Cardiovascular	241 (2.7)

*BMI, Body Mass Index; eGFR, estimated Glomerular Filtration Rate*.

### Prevalence and Clinical Feature of Malnutrition

**Figure 2:**
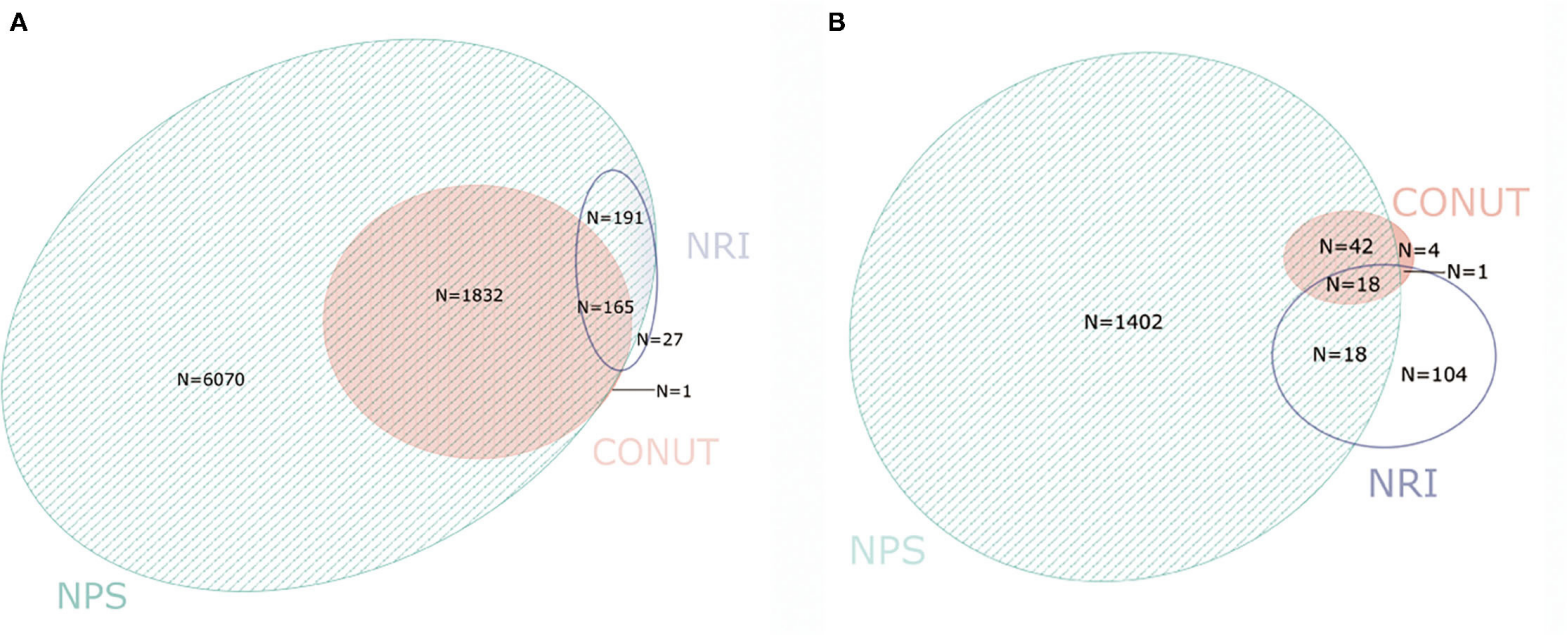
Venn diagram. The numbers in circles indicate the cumulative frequency of malnutrition [any degree **(A)** vs. moderate-severe **(B)**] according to each malnutrition screening tool. The overlapping area of the circles indicate the patient which diagnosed with malnutrition by more than one malnutrition screening tool. CONUT, Controlling Nutritional Status score; NRI, Nutritional Risk Index; NPS, Naples Prognostic Score.

The percentage of malnutrition was 19.9, 3.9, and 82.9% as evaluated by CONUT, NRI, and NPS, respectively. Moreover, the percentage of moderate-to-severe malnutrition varied from 0.7% with CONUT, 2.2% with NRI, and 15.4% with NPS (Table 2). The correlation between three NSTs was weak but significant (CONUT vs. NRI: *r* = 0.137, *P* < 0.01; CONUT vs. NPS: *r* = 0.226, *P* < 0.01; NRI vs. NPS: *r* = 0.063, *P* < 0.01 Figure 2). Patients with malnutrition assessed by any of the three NSTs were older, with lower BMI and hemoglobin level, and worse renal function and more comorbidities than those with normal nutritional status. The former group of patients also had higher all-cause and cardiovascular mortality (Table 3).

**Table 2:** Prevalence of malnutrition according to three nutritional screening tools.

	**Nutritional indices**	**Total (*n* = 9,949)**
CONUT	Normal, *n* (%)	7,950 (79.7)
	Mild, *n* (%)	1,934 (19.4)
	Moderate to severe, *n* (%)	65 (0.7)
NRI	Normal, *n* (%)	9,566 (96.2)
	Mild, *n* (%)	164 (1.6)
	Moderate to severe, *n* (%)	219 (2.2)
NPS	Normal, *n* (%)	1,691 (17.0)
	Mild, *n* (%)	6,700 (67.3)
	Moderate to severe, *n* (%)	1,558 (15.7)

*CONUT, Controlling Nutritional Status score; NRI, Nutritional Risk Index; NPS, Naples Prognostic Score*.

**Table 3:** Comparison of characteristics of study population by different nutritional status.

**Variables**	**COUNT**			**NPS**			**NRI**		
	**Normal**	**Malnutrition**	***P***	**Normal**	**Malnutrition**	***P***	**Normal**	**Malnutrition**	***P***
Age, years	58.5 ± 14.9	64.3 ± 15.4	<0.01	55.5 ± 13.7	60.5 ± 15.4	<0.01	59.3 ± 15.1	67.2 ± 14.8	<0.01
Female gender, *n* (%)	4,226 (53.2)	766 (38.3)	<0.01	1,040 (61.5)	3,952 (47.9)	<0.01	4,785 (50.0)	207 (54.0)	0.14
Ethnicity(white), *n* (%)	3,703 (46.6)	1,047 (52.4)	<0.01	579 (34.2)	4,171 (50.5)	<0.01	4,569 (47.8)	181 (47.3)	0.88
BMI	30.8 ± 7.1	30.0 ± 7.3	<0.01	30.4 ± 6.8	30.7 ± 7.2	0.11	31.0 ± 7.0	21.0 ± 2.8	<0.01
Diabetes mellitus, *n* (%)	1,508 (19.7)	615 (31.9)	<0.01	282 (17.3)	1,841 (23.2)	<0.01	2,064 (22.4)	59 (25.6)	<0.01
Stroke, *n* (%)	492 (6.2)	281 (10.9)	<0.01	79 (4.7)	631 (7.7)	<0.01	657 (6.9)	53 (13.9)	<0.01
Emphysema, *n* (%)	221 (2.8)	100 (5.0)	<0.01	32 (1.9)	289 (3.5)	<0.01	287 (3.0)	34 (8.9)	<0.01
Liver disease, *n* (%)	370 (4.7)	116 (5.8)	0.04	66 (3.9)	420 (5.1)	0.04	467 (4.9)	19 (5.0)	1.00
Malignant tumor, *n* (%)	944 (11.9)	423 (21.2)	<0.01	134 (7.9)	1,233 (14.9)	<0.01	1,293 (13.5)	74 (19.3)	<0.01
Congestive heart failure, *n* (%)	370 (4.7)	238 (12.0)	<0.01	48 (2.8)	560 (6.8)	<0.01	576 (6.0)	32 (8.4)	0.07
Coronary heart disease, *n* (%)	475 (6.0)	288 (14.6)	<0.01	52 (3.1)	711 (8.7)	<0.01	737 (7.8)	26 (6.9)	0.59
Albuminuria, *n* (%)	1,425 (18.1)	524 (21.7)	<0.01	248 (14.7)	1,701 (21.0)	<0.01	1,833 (19.4)	116 (32.0)	<0.01
Hemoglobin (g/L)	14.1 ± 1.5	13.5 ± 1.7	<0.01	14.1 ± 1.4	14.0 ± 1.6	<0.01	14.0 ± 1.6	13.0 ± 1.6	<0.01
eGFR(ml/min/1.73m^2^)	80.9 ± 22.0	71.9 ± 25.5	<0.01	85.3 ± 19.9	77.8 ± 23.4	<0.01	79.5 ± 22.8	70.4 ± 26.7	<0.01
Long-term mortality, (%)									
All-cause	801 (10.1)	453 (22.7)	<0.01	90 (5.3)	1,164 (14.1)	<0.01	1,119 (11.7)	135 (35.2)	<0.01
Cardiovascular	159 (2.2)	82 (5.0)	<0.01	24 (1.5)	217 (3.0)	<0.01	216 (2.5)	25 (9.2)	<0.01

*BMI, Body Mass Index; eGFR, estimated Glomerular Filtration Rate; CONUT, Controlling Nutritional Status score; NRI, Nutritional Risk Index; NPS, Naples Prognostic Score*.

### Malnutrition Score, All-Cause Mortality and Cardiovascular Mortality

**Figure 3:**
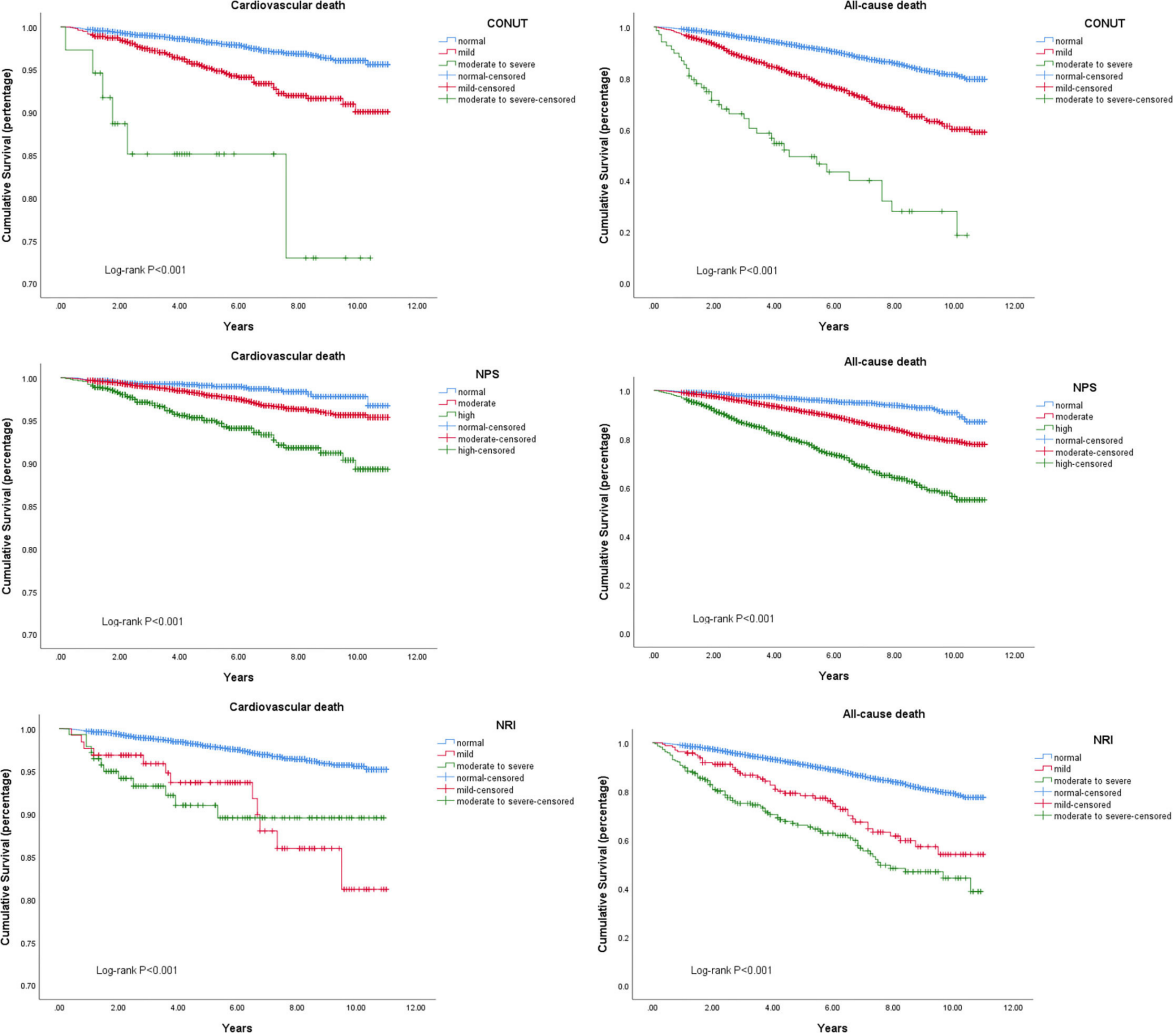
Kaplan–Meier curves of long-term cardiovascular and all-cause mortality for different nutritional status assessed by three nutritional screening tools.

**Figure 4:**
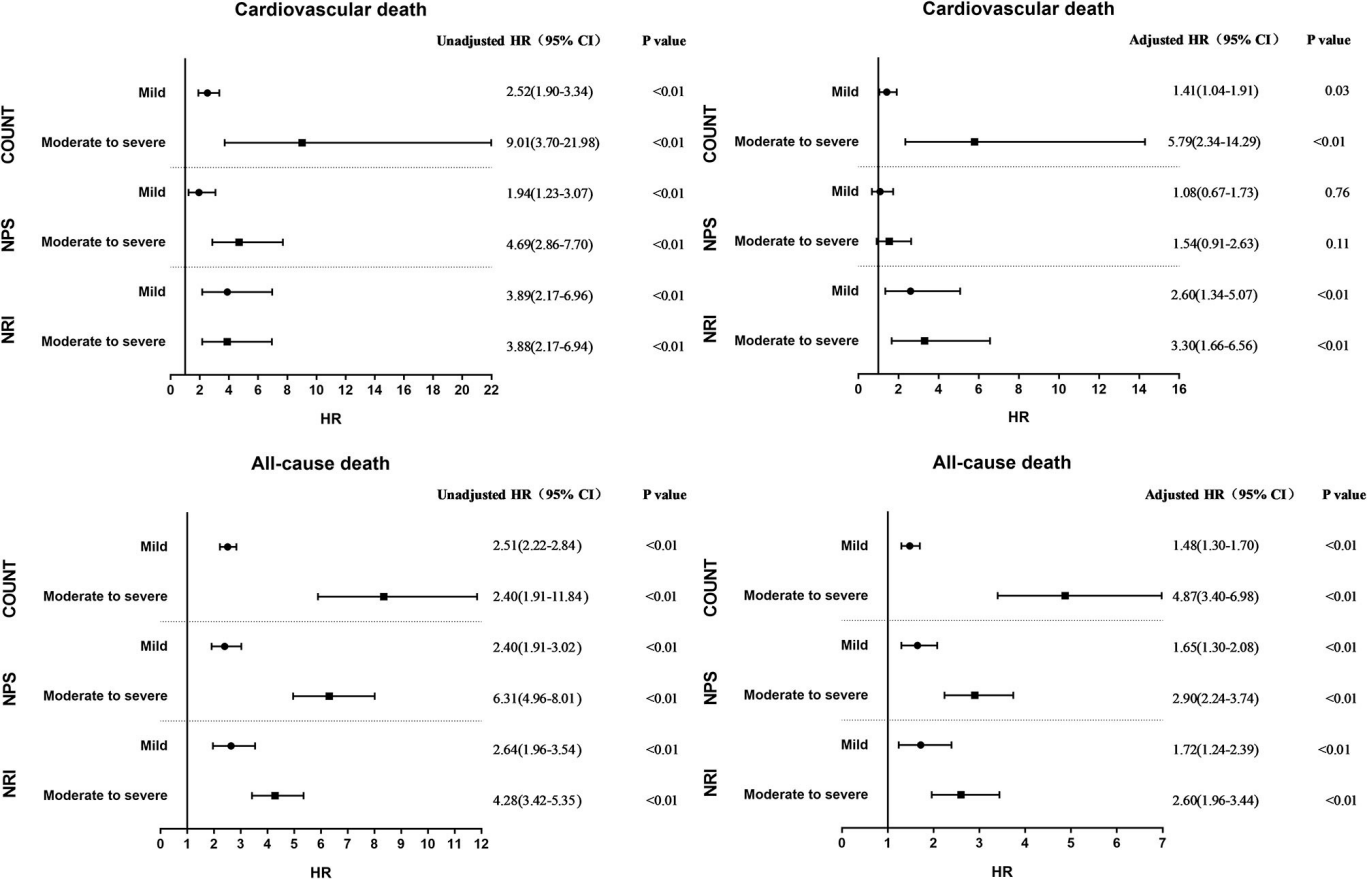
Unadjusted and adjusted hazard ratio for cardiovascular death and all-cause death by degree of malnutrition according to the three nutritional screening tools.

As shown by univariate Cox proportional hazard regression (Tables 4, 5) and Kaplan–Meier survival curves (Figure 3), compared to normal nutritional status, worse nutritional status evaluated by any of three NSTs in both continuous form and categorical form tended to have a higher cardiovascular mortality and all-cause mortality. After adjusting for variables such as age, sex, renal insufficiency, and other diseases that could have influenced long-term mortality in univariate Cox regression analyses (Table 4), malnutrition evaluated by NPS was not associated with higher incidence of cardiovascular death (mild: adjusted HR = 1.08, 95% CI: 0.69–1.73, *P* = 0.76; moderate-to-severe: adjusted HR = 1.54, 95% CI: 0.91–2.63, *P* = 0.11 Table 5; Figure 4), but it was still significant for all-cause mortality prediction (mild: adjusted HR = 1.65, 95% CI: 1.30–2.08, *P* < 0.01; moderate-to-severe: adjusted HR = 2.90, 95% CI: 2.24–3.74, *P* < 0.01). Expect for NPS, both CONUT and NRI could independently predict cardiovascular mortality (adjusted HR (95%CI) for mild and moderate-to-severe degree of malnutrition, respectively: 1.41 (1.04–1.91) and 5.79 (2.34–14.29) for CONUT; 2.60 (1.34–5.07) and 3.30 (1.66–6.56) for NRI, all *P* < 0.05) and all-cause mortality (adjusted HR (95%CI) for mild and moderate-to-severe degree of malnutrition, respectively: 1.48 (1.30–1.70) and 4.87 (3.40–6.98) for CONUT; 1.72 (1.24–2.39) and 2.60 (1.96–3.44) for NRI, all *P* < 0.01) and further adjustment for educational level, family income, current smoking, alcohol intake, and diet health also showed a similar result (Supplementary Tables 1A,B). By further stratified by age, chronic disease, and blood pressure control, NRI could only independently predict cardiovascular mortality and all-cause mortality in hypertensive patient with aged over 60 or comorbidities (Supplementary Tables 2A,B) and had advantages on predicting cardiovascular mortality among hypertensive patients with controlled blood pressure (Supplementary Table 2C).

**Table 4:** Univariate Cox regression for long-term mortality.

**Variables**	**Cardiovascular death**			**All cause death**		
	**HR**	**95%CI**	***P***	**HR**	**95%CI**	***P***
Age	1.10	1.09–1.12	<0.01	1.08	1.08–1.09	<0.01
Female gender	0.59	0.46–0.77	<0.01	0.79	0.70–0.88	<0.01
White race	1.79	1.38–2.32	<0.01	1.62	1.45–1.82	<0.01
BMI (continuous)	0.95	0.93–0.97	<0.01	0.95	0.94–0.96	<0.01
Current smoke	1.04	0.75–1.43	0.83	1.08	0.93–1.24	0.31
Diabetes mellitus	1.74	1.32–2.30	<0.01	1.61	1.42–1.82	<0.01
Stroke	3.28	2.34–4.60	<0.01	2.97	2.56–3.44	<0.01
Emphysema	3.44	2.13–5.57	<0.01	3.64	3.00–4.41	<0.01
Liver disease	0.72	0.36–1.46	0.34	1.21	0.94–1.54	0.14
Malignant tumor	1.95	1.43–2.65	<0.01	2.07	1.81–2.36	<0.01
Congestive heart failure	7.12	5.32–9.52	<0.01	3.69	3.18–4.29	<0.01
Coronary heart disease	4.49	3.34–6.04	<0.01	2.48	2.13–2.89	<0.01
Albuminuria	3.45	2.65–4.49	<0.01	3.16	2.81–3.54	<0.01
Hemoglobin	0.83	0.77–0.90	<0.01	0.81	0.78–0.84	<0.01
eGFR <60 ml/min/1.73 m^2^	4.28	3.33–5.52	<0.01	3.55	3.18–3.97	<0.01
CONUT (continuous)	1.54	1.41–1.68	<0.01	1.51	1.46–1.58	<0.01
NRI (continuous)	0.96	0.95–0.96	<0.01	0.96	0.95–0.96	<0.01
NPS (continuous)	1.72	1.52–1.94	<0.01	1.71	1.62–1.81	<0.01

*BMI, Body Mass Index; eGFR, estimated Glomerular Filtration Rate; CONUT, Controlling Nutritional Status score; NRI, Nutritional Risk Index; NPS, Naples Prognostic Score*.

**Table 5:** Univariate and multivariate Cox regression of three nutritional screening tools for cardiovascular death and all-cause death.

**Variables**	**Cardiovascular death**				**All-cause death**			
	**Unadjusted**		Adjusted^*^		**Unadjusted**		Adjusted^*^	
	**HR (95%CI)**	***P***	**HR (95%CI)**	***P***	**HR (95%CI)**	***P***	**HR (95%CI)**	***P***
CONUT Normal	/		/		/		/	
Mild	2.52 (1.90–3.34)	<0.01	1.41 (1.04–1.91)	0.03	2.51 (2.22–2.84)	<0.01	1.48 (1.30–1.70)	<0.01
Moderate to severe	9.01 (3.70–21.98)	<0.01	5.79 (2.34–14.29)	<0.01	8.35 (5.89–11.84)	<0.01	4.87 (3.40–6.98)	<0.01
NPS Normal	/		/		/		/	
Mild	1.94 (1.23–3.07)	<0.01	1.08 (0.67–1.73)	0.76	2.40 (1.91–3.02)	<0.01	1.65 (1.30–2.08)	<0.01
Moderate to severe	4.69 (2.86–7.70)	<0.01	1.54 (0.91–2.63)	0.11	6.31 (4.96–8.01)	<0.01	2.90 (2.24–3.74)	<0.01
NRI Normal	/		/		/		/	
Mild	3.89 (2.17–6.96)	<0.01	2.60 (1.34–5.07)	<0.01	2.64 (1.96–3.54)	<0.01	1.72 (1.24–2.39)	<0.01
Moderate to severe	3.88 (2.17–6.94)	<0.01	3.30 (1.66–6.56)	<0.01	4.28 (3.42–5.35)	<0.01	2.60 (1.96–3.44)	<0.01

* *Adjusted with age, sex, white race, BMI, diabetes mellitus, stroke, emphysema, malignant tumor, congestive heart failure, coronary heart disease, hemoglobin, eGFR (estimated Glomerular Filtration Rate), abuminuria*.

## Discussion

In this study, we reported the prevalence and prognostic value of malnutrition among 9,949 hypertensive patients in a community setting by three NSTs. Our research showed that malnutrition as assessed by NSTs was common among hypertensive patients and associated with both cardiovascular and all-cause mortality.

Hypertension is considered a nutritional factor-related disease (16, 17). Few studies have analyzed the prevalence of malnutrition in hypertensive patients. Sun et al. (18) reported that the prevalence of malnutrition in elderly patients with hypertension was 52.4 and 27.1% for mild malnutrition and moderate-to-severe malnutrition, respectively, using the CONUT scoring system in a cohort of 336 patients aged ≥80 years. In our study of hypertensive patients, the percentage of individuals with malnutrition assessed by CONUT, NRI, and NPS was 19.9, 3.9, and 82.9%, respectively. Moreover, 0.7, 2.2, and 15.4% of individuals with hypertension were classified as having moderate-to-severe malnutrition assessed by CONUT, NRI, and NPS, respectively. The remarkable difference in malnutrition prevalence among the three screening tools might because of different parameters included or different thresholds for the same parameter. The poor concordance in identifying malnutrition among the three screening tools suggest that they are not interchangeable. The nutritional status of hypertensive patients evaluated by CONUT and NPS suggest that malnutrition was quite common among these patients. On the one hand, it might be owing to the physiological interrelationship between hypertension and inflammation (4, 19). Previous studies have reported that hypertensive patients had higher plasma concentrations of proinflammatory cytokines and acute phase proteins (20). The activation of inflammatory pathways might increase the catabolic demands and result in malnutrition. On the other hand, vitamin D deficiency was prevalent among malnourished patients (21) and its deficiency was highly associated with incidence of metabolic syndrome (22) and cardiovascular disease including hypertension (23), while vitamin D could act on endothelial cells and smooth muscle cells to regulate blood pressure (24).

The relationship between nutritional status and prognosis has been confirmed in some cardiovascular diseases. In a study including 5,062 acute coronary syndrome patients with a median age of 66.2 years, multivariate cox proportional hazard regression analysis indicated that malnutrition assessed by CONUT and NRI was an independent factor for all-cause mortality and cardiovascular events (25). Another study involving 336 elderly hypertensive patients confirmed that poor nutritional status assessed by the CONUT score was significantly associated with all-cause mortality (18). In the present study, malnutrition evaluated by different NSTs was also significantly associated with both cardiovascular and all-cause mortality in hypertensive patients. The possible underlying mechanism might be explained by the following facts. First, tumor necrosis factor-alpha (TNF-α), a key inflammatory mediator (26), was found to be higher in patients with moderate-to-severe malnutrition. The underlying inflammation process could involve the pathogenesis and progress of some cardiovascular diseases (27, 28) such as coronary artery disease (29, 30) and heart failure (31), thereby supplementing the cardiovascular risk brought on by hypertension (32) and finally leading to cardiovascular events. Second, as mentioned previously, malnutrition patient always comorbidity with vitamin D deficiency (21, 33). The deficiency of vitamin D was reported associated with higher risk of uncontrolled BP in hypertensive patients (34) which was associated premature vascular death and CVD mortality (35). Third, all three NSTs include serum albumin as a parameter, while hypoalbuminemia was confirmed to be associated with extremely poor prognosis and cardiac cachexia (36, 37).

Given that malnutrition assessed by NSTs is now a common occurrence among hypertensive patients and proved to be associated with higher cardiovascular and all-cause mortality, the question remains, which NST is suitable for clinicians to identify hypertensive patients with malnutrition. Our study showed that NPS was not suitable for predicting long-term cardiovascular mortality, while nutritional status evaluated by NPS was significantly associated with all-cause mortality but not cardiovascular mortality after adjustment for potential confounding factors. This phenomenon might be due to the threshold setting of its parameters. In the NPS scoring system (14), patients with serum albumin <40 g/L were considered to have malnutrition, while the threshold for serum albumin was 35 g/L in the CONUT scoring system (12), which led to its poor ability to distinguish patients with and without malnutrition. Although nutritional status assessed by NRI was significantly associated with both all-cause mortality and cardiovascular mortality, it showed poorer performance than CONUT in differentiating patients with mild and moderate-to-severe malnutrition in terms of mortality risk; this could be explained by the small number of patients with malnutrition evaluated by NRI. Moreover, NRI was recommended for identify malnutrition among elderly hypertensive patients or with comorbidities, while the NRI had advantages on predicting both cardiovascular mortality and all-cause mortality among elderly hypertensive patients or with comorbidities. Overall, CONUT was a more suitable NST than NPS and NRI to identify malnutrition among all hypertensive patients.

## Limitation

There were some limitations to this study that should be noted. First, the proportion of patients who were classified with moderate-to-severe malnutrition was low leading to the limited value of this study for those hypertensive patients with extremely poor nutritional status. Second, because of the retrospective study design, our findings should be interpreted with caution. Finally, although the models were adjusted for potential risk factors using multiple regression analysis techniques, there may have been some residual confounding factors.

## Conclusion

Malnutrition evaluated by NSTs was common among hypertensive patients and was closely associated with both long-term cardiovascular mortality and all-cause mortality. Clinicians should make additional efforts for the early identification and management of malnutrition.

## Data Availability Statement

The original contributions presented in the study are included in the article/Supplementary Material, further inquiries can be directed to the corresponding author/s.

## Ethics Statement

The studies involving human participants were reviewed and approved by Ethics Review Committee of NCHS of the Centers for Disease Control and Prevention. The patients/participants provided their written informed consent to participate in this study. Written informed consent was obtained from the individual(s), and minor(s)' legal guardian/next of kin, for the publication of any potentially identifiable images or data included in this article.

## Author Contributions

Z-wY, X-bW, D-qY, and J-yC contributed to the conception or design of the study. Z-wY contributed to the acquisition, analyses, and interpretation of data. Z-wY, X-bW, and B-qF drafted the manuscript. D-qY revised the manuscript critically, had all access to the data, and is responsible for the overall content as guarantor. All authors contributed to refinement of the study protocol and approved the final manuscript.

## Funding

This work was supported by grants from National Natural Science Foundation of China (Grant No. 82002014), Natural Science Foundation of Guangdong Province (Grant No. 2021A1515010107), and Science and Technology Projects of Guangzhou (Grant No. 201903010097). The funders had no role in the study design, data collection and analysis, decision to publish, nor preparation of the manuscript.

## Conflict of Interest

The authors declare that the research was conducted in the absence of any commercial or financial relationships that could be construed as a potential conflict of interest.

## Publisher's Note

All claims expressed in this article are solely those of the authors and do not necessarily represent those of their affiliated organizations, or those of the publisher, the editors and the reviewers. Any product that may be evaluated in this article, or claim that may be made by its manufacturer, is not guaranteed or endorsed by the publisher.
