# Corrigendum: Prevalence and Prognostic Significance of Malnutrition in Hypertensive Patients in a Community Setting

**DOI:** 10.3389/fnut.2022.903202

**Published:** 2022-04-21

**Authors:** Zhi-wen Yang, Xue-biao Wei, Bing-qi Fu, Ji-yan Chen, Dan-qing Yu

**Affiliations:** ^1^Shantou University Medical College, Shantou, China; ^2^Division of Cardiology, Guangdong Cardiovascular Institute, Guangdong Provincial Key Laboratory of Coronary Heart Disease Prevention, Guangdong Provincial People's Hospital, Guangdong Academy of Medical Sciences, Guangzhou, China; ^3^Division of Geriatrics Intensive Medicine, Guangdong Provincial Geriatrics Institute, Guangdong Provincial People's Hospital, Guangdong Academy of Medical Sciences, Guangzhou, China

**Keywords:** malnutrition, hypertension, Controlling Nutritional Status (CONUT) score, Nutritional Risk Index (NRI), Naples Prognostic Score, cardiovascular mortality, all-cause mortality

In the published article, there was an error in affiliation 1. Instead of “Department of Clinical Medicine, Shantou University Medical College, Shantou, China,” it should be “Shantou University Medical College, Shantou, China.”

In the published article, there was also an error in affiliation 2. Instead of “Guangdong Provincial Key Laboratory of Coronary Heart Disease Prevention, Division of Cardiology, Guangdong Cardiovascular Institute, Guangdong Provincial People's Hospital, Guangdong Academy of Medical Sciences, Guangzhou, China,” it should be “Division of Cardiology, Guangdong Cardiovascular Institute, Guangdong Provincial Key Laboratory of Coronary Heart Disease Prevention, Guangdong Provincial People's Hospital, Guangdong Academy of Medical Sciences, Guangzhou, China.”

In the published article, there was also an error in affiliation 3. Instead of “Division of Critical Care Medicine, Geriatrics Institute, Guangdong Provincial People's Hospital, Guangdong Academy of Medical Sciences, Guangzhou, China,” it should be “Division of Geriatrics Intensive Medicine, Guangdong Provincial Geriatrics Institute, Guangdong Provincial People's Hospital, Guangdong Academy of Medical Sciences, Guangzhou, China.”

The authors apologize for this error and state that this does not change the scientific conclusions of the article in any way. The original article has been updated.

## Publisher's Note

All claims expressed in this article are solely those of the authors and do not necessarily represent those of their affiliated organizations, or those of the publisher, the editors and the reviewers. Any product that may be evaluated in this article, or claim that may be made by its manufacturer, is not guaranteed or endorsed by the publisher.

