# Supplementary material for: Prevalence and Prognostic Significance of Malnutrition in Hypertensive Patients in a Community Setting
Source: Front Nutr. 2022 Feb 23;9:822376. doi: 10.3389/fnut.2022.822376 (PMC8905503; doi:10.3389/fnut.2022.822376)
Supplement: Supplementary file 1 [file Data_Sheet_1.docx]

**Supplementary table 1a. Multivariate Cox regression of 3 nutritional screening tools for cardiovascular death and all-cause death**

| **Variables** | Cardiovascular death  (Adjusted for model 2) | | All-cause death  (Adjusted for model 2) | |
| --- | --- | --- | --- | --- |
|  | **HR (95%CI)** | **P** | **HR (95%CI)** | **P** |
| CONUT Normal  Mild  Moderate to severe | /  1.44(1.05-1.98)  4.60(1.82-11.64) | 0.02  <0.01 | /  1.57(1.37-1.81)  4.92(3.37-7.19) | <0.01  <0.01 |
| NPS Normal  Mild  Moderate to severe | /  1.12(0.68-1.83)  1.71(0.98-2.97) | 0.66  0.06 | /  1.64(1.28-2.09)  2.92(2.24-3.81) | <0.01  <0.01 |
| NRI Normal  Mild  Moderate to severe | /  3.31(1.68-6.51)  3.08(1.62-5.85) | <0.01  <0.01 | /  1.84(1.30-2.60)  2.59(1.93-3.50) | <0.01  <0.01 |

Model 2 included: educational level, family income, smoking, alcohol intake, age, sex, white race, BMI, diabetes mellitus, stroke, emphysema, malignant tumor, congestive heart failure, coronary heart disease, hemoglobin, eGFR(estimated glomerular filtration rate) and abuminuria.

**Supplementary table 1b. Multivariate Cox regression of 3 nutritional screening tools for cardiovascular death and all-cause death**

| **Variables** | Cardiovascular death  (Adjusted for model 3) | | All-cause death  (Adjusted for model 3) | |
| --- | --- | --- | --- | --- |
|  | **HR (95%CI)** | **P** | **HR (95%CI)** | **P** |
| CONUT Normal  Mild  Moderate to severe | /  1.44(1.05-1.98)  4.60(1.82-11.64) | 0.02  <0.01 | /  1.57(1.37-1.81)  4.92(3.37-7.19) | <0.01  <0.01 |
| NPS Normal  Mild  Moderate to severe | /  1.12(0.69-1.84)  1.72(0.99-2.99) | 0.64  0.05 | /  1.64(1.28-2.09)  2.92(2.24-3.81) | <0.01  <0.01 |
| NRI Normal  Mild  Moderate to severe | /  3.31(1.68-6.51)  3.08(1.62-5.85) | <0.01  <0.01 | /  1.84(1.30-2.60)  2.59(1.93-3.50) | <0.01  <0.01 |

Model 3 included: diet health, educational level, family income, smoking, alcohol intake, age, sex, white race, BMI, diabetes mellitus, stroke, emphysema, malignant tumor, congestive heart failure, coronary heart disease, hemoglobin, eGFR(estimated glomerular filtration rate) and abuminuria.

**Supplementary table 2a. Multivariate Cox regression of 3 nutritional screening tools for cardiovascular death and all-cause death stratified by age**

| Variables | Cardiovascular death | | | | |  | All-cause death | | | | |
| --- | --- | --- | --- | --- | --- | --- | --- | --- | --- | --- | --- |
|  | Age＜60 years old | |  | Age≥60 years old | |  | Age＜60 years old | |  | Age≥60 years old | |
|  | HR (95%CI) | P |  | HR (95%CI) | P |  | HR (95%CI) | P |  | HR (95%CI) | P |
| CONUT Normal  Mild  Moderate to severe | /  1.20(0.44-3.25)  13.17(2.72-63.71) | 0.71  <0.01 |  | /  1.55(1.12-2.14)  2.07(0.63-6.82) | 0.01  0.23 |  | /  2.04(1.41-2.95)  14.60(6.76-31.52) | <0.01  <0.01 |  | /  1.49(1.29-1.71)  3.60(2.39-5.42) | <0.01  <0.01 |
| NPS Normal  Mild  Moderate to severe | /  1.03(0.38-2.81)  1.24(0.34-4.67) | 0.95  0.72 |  | /  1.13(0.66-1.93)  1.79(0.99-3.24) | 0.65  0.05 |  | /  1.75(1.10-2.80)  2.99(1.72-5.19) | 0.02  <0.01 |  | /  1.66(1.26-2.19)  3.02(2.25-4.05) | 0.02  <0.01 |
| NRI Normal  Mild  Moderate to severe | /  3.89(0.59-19.99)  0.01* | 0.17  0.91 |  | /  2.29(1.10-4.76)  3.90(1.94-7.82) | 0.02  0.01 |  | /  1.24(0.37-4.14)  0.74(0.16-3.31) | 0.72  0.69 |  | /  1.74(1.24-2.45)  2.63(1.97-3.51) | <0.01  <0.01 |

Adjusted with sex, white race, BMI, diabetes mellitus, stroke, emphysema, malignant tumor, congestive heart failure, coronary heart disease, hemoglobin, eGFR(estimated glomerular filtration rate),abuminuria.

*The confidence interval: 5.0×10^-58^ to 1.6×10^51^

**Supplementary table 2b. Univariate and multivariate Cox regression of 3 nutritional screening tools for cardiovascular death and all-cause death** **stratified by chronic disease**

| Variables | Cardiovascular death | | | | |  | All-cause death | | | | |
| --- | --- | --- | --- | --- | --- | --- | --- | --- | --- | --- | --- |
|  | With chronic disease | |  | Without chronic disease | |  | With chronic disease | |  | Without chronic disease | |
|  | HR (95%CI) | P |  | HR (95%CI) | P |  | HR (95%CI) | P |  | HR (95%CI) | P |
| CONUT Normal  Mild  Moderate to severe | /  1.57(1.10-2.34)  4.10(1.28-13.13) | 0.01  0.02 |  | /  1.63(1.01-2.65)  13.6(3.29-56.4) | 0.04  <0.01 |  | /  1.56(1.34-1.82)  4.99(3.36-7.39) | <0.01  <0.01 |  | /  1.62(1.31-2.02)  5.60(2.46-12.78) | <0.01  <0.01 |
| NPS Normal  Mild  Moderate to severe | /  1.22(0.63-2.36)  2.11(1.04-4.27) | 0.56  0.04 |  | /  0.95(0.53-1.73)  1.79(0.90-3.56) | 0.88  0.10 |  | /  1.60(1.14-2.24)  3.22(2.28-4.56) | <0.01  <0.01 |  | /  1.55(1.14-2.10)  2.51(1.77-3.56) | <0.01  <0.01 |
| NRI Normal  Mild  Moderate to severe | /  4.28(2.15-8.51)  2.85(1.24-6.54) | <0.01  <0.01 |  | /  0.92(0.22-3.93)  2.26(0.79-6.45) | 0.91  0.13 |  | /  1.83(1.23-2.73)  2.98(2.14-4.16) | <0.01  <0.01 |  | /  1.23(0.72-2.12)  1.74(1.06-2.83) | 0.45  0.03 |

Adjusted with age, sex, white race, BMI, hemoglobin, eGFR(estimated glomerular filtration rate),abuminuria.

**Supplementary table 2c. Multivariate Cox regression of 3 nutritional screening tools for cardiovascular death and all-cause death stratified by blood pressure control**

| Variables | Cardiovascular death | | | | |  | All-cause death | | | | |
| --- | --- | --- | --- | --- | --- | --- | --- | --- | --- | --- | --- |
|  | BP＜140/90mmHg | |  | BP≥140/90mmHg | |  | BP＜140/90mmHg | |  | BP≥140/90mmHg | |
|  | HR (95%CI) | P |  | HR (95%CI) | P |  | HR (95%CI) | P |  | HR (95%CI) | P |
| CONUT Normal  Mild  Moderate to severe | /  1.61(1.04-2.49)  3.31(0.77-14.25) | 0.03  <0.01 |  | /  1.45(0.94-2.22)  7.22(2.23-23.35) | 0.09  <0.01 |  | /  1.74(1.44-2.10)  6.26(4.03-9.70) | <0.01  <0.01 |  | /  1.42(1.17-1.72)  3.76(1.93-7.33) | <0.01  <0.01 |
| NPS Normal  Mild  Moderate to severe | /  1.29(0.54-3.05)  2.05(0.82-5.16) | 0.56  0.13 |  | /  1.10(0.57-1.78)  1.48(0.75-2.91) | 0.97  0.25 |  | /  3.72(2.46-5.60)  2.99(1.72-5.19) | <0.01  <0.01 |  | /  1.53(1.13-2.06)  2.62(1.88-3.65) | <0.01  <0.01 |
| NRI Normal  Mild  Moderate to severe | /  4.76(2.04-11.12)  3.42(1.24-9.46) | <0.01  0.02 |  | /  1.80(0.68-4.71)  3.46(1.41-8.47) | 0.23  0.03 |  | /  1.64(0.96-2.80)  3.29(2.28-4.74) | 0.07  <0.01 |  | /  2.13(1.46-3.11)  2.26(1.61-3.18) | <0.01  <0.01 |

Adjusted with sex, white race, BMI, diabetes mellitus, stroke, emphysema, malignant tumor, congestive heart failure, coronary heart disease, hemoglobin, eGFR(estimated glomerular filtration rate),abuminuria.
